# Senescence in the ageing skin: a new focus on mTORC1 and the lysosome

**DOI:** 10.1111/febs.17281

**Published:** 2024-09-26

**Authors:** Phineas Smith, Bernadette Carroll

**Affiliations:** ^1^ School of Biochemistry University of Bristol UK

**Keywords:** ageing, lysosome, mTORC1, senescence, skin

## Abstract

Ageing is defined as the progressive loss of tissue function and regenerative capacity and is caused by both intrinsic factors i.e. the natural accumulation of damage, and extrinsic factors i.e. damage from environmental stressors. Cellular senescence, in brief, is an irreversible exit from the cell cycle that occurs primarily in response to excessive cellular damage, such as from ultraviolet (UV) exposure and oxidative stress, and it has been comprehensively demonstrated to contribute to tissue and organismal ageing. In this review, we will focus on the skin, an organ which acts as an essential protective barrier against injury, insults, and infection. We will explore the evidence for the existence and contribution of cellular senescence to skin ageing. We discuss the known molecular mechanisms driving senescence in the skin, with a focus on the dysregulation of the master growth regulator, mechanistic Target of Rapamycin Complex 1 (mTORC1). We explore the interplay of dysregulated mTORC1 with lysosomes and how they contribute to senescence phenotypes.

## Graphical abstract

The accumulation of senescent cells in the skin contributes to age‐related phenotypes including extracellular matrix (ECM) degradation, chronic inflammation, and epidermal thinning. These changes impair the skin's fundamental role as a barrier against insult, injury, and infection. Here we highlight the underappreciated role of mTORC1 signalling and the lysosome in supporting senescence phenotypes in the skin, including the inflammatory senescence‐associated secretory phenotype (SASP).

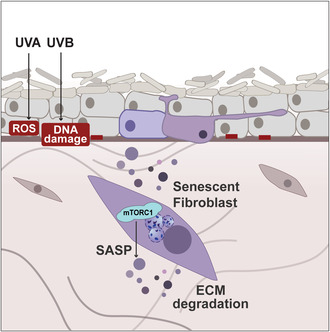

**Abbreviations**
4E‐BP1: eIF4E‐binding protein 1
ABCA1: ATP‐binding cassette transporter A1
BCL‐2: B‐cell lymphoma‐2
BM: basement membrane
CCF: cytoplasmic chromatin fragments
CD4/8: cluster of differentiation 4/8
CDKI: cyclin‐dependent kinase inhibitors
CMA: chaperone‐mediated autophagy
COL17A1: collagen, type XVII, alpha 1
CXCL: C‐X‐C motif ligand
CXCR: C‐X‐C motif chemokine receptor 3
DDR: DNA damage response
ECM: extracellular matrix
EDJ: epidermal‐dermal junction
ER: endoplasmic reticulum
Erk: extracellular signal regulated kinase
ESCRT: endosomal sorting complex required for transport
EV: extracellular vesicle
HF: hair follicles
HLA‐E: human leukocyte antigen E
IFE: interfollicular epidermis
IFN‐γ: interferon‐γ
IGF‐1/‐1R: insulin‐like growth factor‐1/‐1 receptor
IKK: IκB kinase
IL: interleukin
IP‐10: interferon gamma‐induced protein 10
KGA: kidney‐type glutaminase
Lamp: lysosomal‐associated membrane protein
LLOMe: l‐leucyl‐l‐leucine methyl ester
MAPKAPK2: MAPK‐activated protein kinase 2
MMP: matrix metalloproteinases
MSH: melanocyte stimulating hormone
mtDNA: mitochondrial DNA
mTORC1: mechanistic target of rapamycin complex 1
NF‐κβ: nuclear factor‐kappa B
NLRP3: nucleotide‐binding oligomerization domain, leucine‐rich repeat and lyrin domain containing
PGC‐1: peroxisome proliferator‐activated receptor gamma coactivator 1
PTEN: phosphatase and tensin homologue
ROS: reactive oxygen species
SAASP: skin ageing‐associated secreted protein profile
SAHF: senescence‐associated heterochromatin foci
SASP: senescence‐associated secretory phenotype
SDF‐1: stromal cell‐derived factor 1
Sen‐β‐Gal: senescence‐associated β‐galactosidase
TAF/TIF: telomere‐associated/telomere‐induced DNA damage foci
TFEB/TFE3: transcription factor EB/E3
TIMP: tissue inhibitor of metalloproteinase
UVA/B: ultraviolet A/B
V‐ATPase: vacuolar‐type ATPase
ZFP36L1: zinc finger protein 36, C3H type‐like 1

## Senescence – an overview

The observation that differentiated human diploid cells have a finite lifespan was first described by Hayflick and Moorhead in 1961 [1]. In a process we now call senescence, cells at the end of their replicative lifespan, or more commonly in response to extrinsic stressors, can irreversibly exit the cell cycle. Cellular senescence is recognised primarily as a tumour‐suppressor mechanism to prevent proliferation after potentially transformative insults, such as UV irradiation and oxidative damage, and has also been shown to play important physiological roles during development and wound healing [2]. However, the accumulation of senescence negatively impacts tissues via a variety of mechanisms, for example through secretion of inflammatory factors, and their significance in ageing is exemplified by the observation that senescent cell clearance in mouse models leads to an extension in their healthy lifespan (‘healthspan’) [3, 4]. Senescence is therefore considered by some evolutionary biologists to be an example of antagonistic pleiotropy, where it is beneficial early on in the lifetime of an organism, while detrimental as it accumulates in tissues with age [5]. Senescent cells accumulate in many bodily tissues with age, including the liver, pancreas, and kidney [6, 7]. Although both the adaptive and innate immune system drive the clearance of senescent cells, as shown by *in vivo* studies of senescence in liver tissue [8, 9], the age‐dependent accumulation of senescent cells may be a result of a decline in the efficacy of the immune system—an idea supported by the increased senescent load seen in immune‐deficient ageing mouse models [10].

**Fig. 1:**
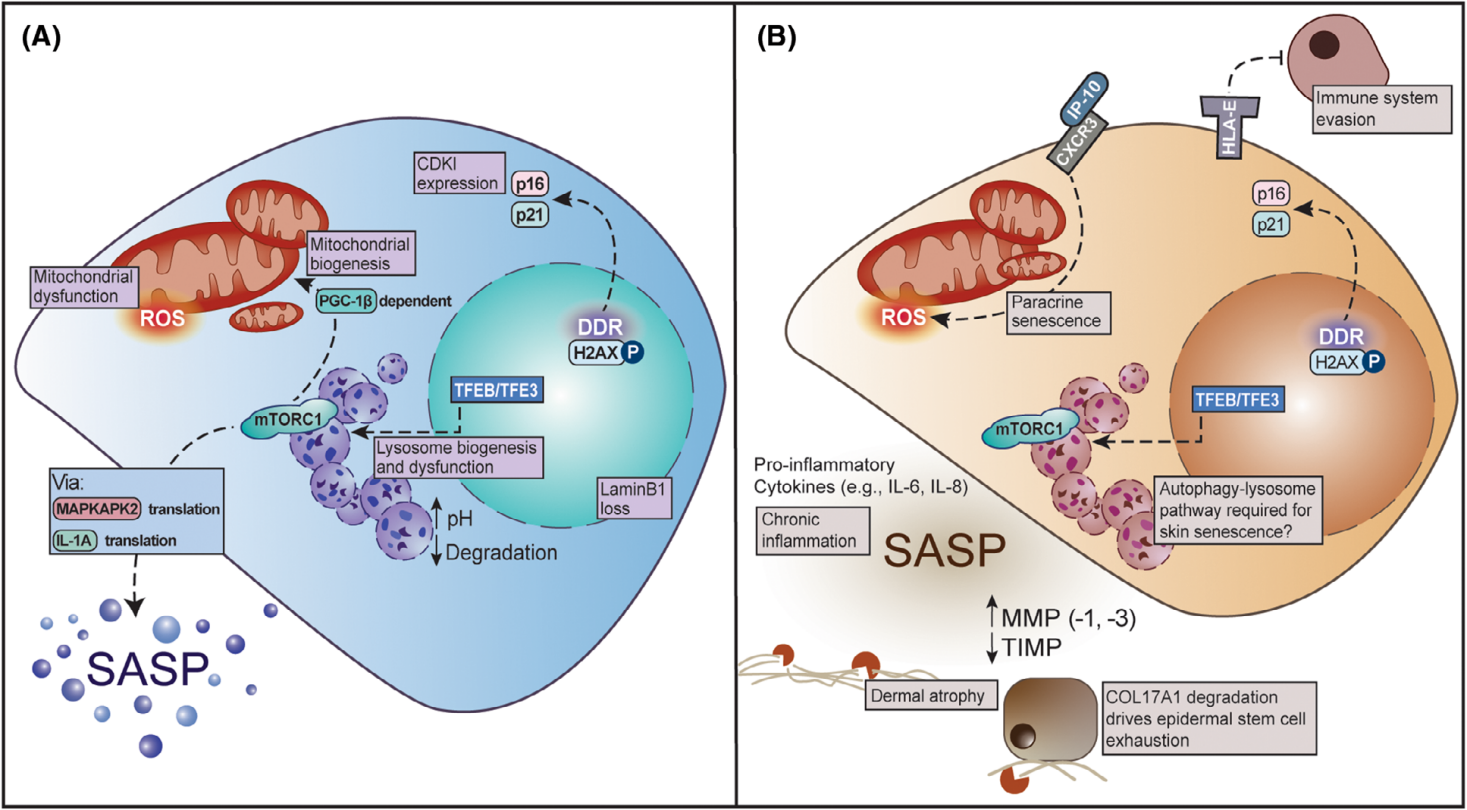
Cellular senescence in the skin. (A) The hallmarks of cellular senescence. In the nucleus, lamina component Lamin B1 is lost [14], and the DNA Damage Response (DDR) is marked by phosphorylation of histone H2AX. Expression of Cyclin‐dependent kinase inhibitors (CDKIs), e.g., p16/p21, halt cell cycle progression [11]. Senescent cells show biogenesis of mitochondria (e.g., through mTORC1‐driven PGC‐1β activity [15]) and lysosomes (through transcription factors TFEB and TFE3 [16]). Both organelles show senescence‐associated dysfunction such as increased mitochondrial ROS production [15], and increased luminal pH and diminished hydrolytic capacity of lysosomes (indicated by up and down arrows respectively) [16]. Expression of the Senescence‐Associated Secretory Phenotype (SASP) is supported by mTORC1 activity [17]. (B) Senescence phenotypes relevant to skin ageing: Paracrine skin cell senescence can be driven through IP‐10 secretion by senescent melanocytes [18]. Extracellular matrix atrophy is enhanced by senescence through increased matrix metalloproteinase (MMP) expression and a concomitant decrease in tissue inhibitors of metalloproteinases (TIMP) expression in the SASP (indicated by up and down arrows respectively) [19]. Stem cell exhaustion in the epidermis via MMP‐driven hemidesmosome degradation (specifically collagen type XVII, COL17A1) may be enhanced by senescence [20]. Inflammation is driven by increased cytokine expression in the SASP (e.g., interleukins, IL‐6/‐8) [21], and immune system evasion is supported by altered histocompatibility complex expression (e.g., human leukocyte antigen E (HLA‐E)) [22]. Dashed lines represent signalling cascades.

Senescence acquisition and maintenance are associated with several well‐described hallmarks, summarised in Fig. 1A and well‐reviewed in Hernandez‐Segura *et al*. [11]. While few hallmarks are universal, they are collectively utilised to accurately and consistently identify senescence *in vitro* and *in vivo*. One of the most notable hallmarks of senescence is increased DNA damage, measured by DNA damage foci decorated with phosphorylated histone H2AX, and detection of telomere‐associated or telomere‐induced DNA damage foci (TAF/TIF) [12]. The DNA Damage Response (DDR) can occur in response to critical telomere shortening, or in a telomere length‐independent manner from direct damage (e.g., ionising radiation, oxidative stress), and via replicative stress during oncogene activation [2, 13]. Activation of the DDR promotes activation of cell cycle arrest signalling which culminates in the expression of cyclin‐dependent kinase inhibitors (CDKI). Two CDKI comprehensively shown to be robust markers of senescence *in vivo* and *in vitro* are p21^CIP1/WAF1^ and p16^INK4A^ (referred to as p21 and p16, respectively). Yh

Senescence is further characterised by genomic instability, including loss of the nuclear lamina component LaminB1 [14], and the presence of cytoplasmic chromatin fragments [11, 23, 24]. Senescence‐associated heterochromatin foci (SAHF) have also been observed, primarily *in vitro* in oncogene‐induced senescence models, and linked to transcriptionally suppressing the progression of the cell cycle [12]. Senescent cells also acquire resistance to apoptosis which is driven by constitutive expression of the antiapoptotic BCL‐2 family of proteins [25].

Despite their non‐proliferative state, senescent cells are characterised by pro‐growth, anabolic phenotypes, supported in part by the simultaneous activation of catabolism, such as the autophagy‐lysosomal pathway. These pro‐growth changes include an increase in cell size, expression of a well‐characterised Senescence‐Associated Secretory Phenotype (referred to as SASP), and an increase in lysosome and mitochondria biogenesis. Increased lysosomal content has long been the gold‐standard for senescence identification *in vitro* and *in vivo*, via staining for senescence‐associated β‐galactosidase activity (Sen‐β‐Gal staining), a lysosomal hydrolase [26]. Although the cellular content of lysosomes and mitochondria is increased, dysfunction of both organelles is well documented in senescence [15, 16, 26, 27, 28]. Many of these pro‐growth phenotypes have been widely attributed to the activation of the mechanistic Target of Rapamycin Complex 1 (mTORC1) signalling pathway and the acute mTORC1 inhibitor, rapamycin, reduces senescence phenotypes *in vitro* and *in vivo* [29, 30, 31].

### mTORC1 activity supports senescence‐associated phenotypes

mTORC1 is an evolutionarily conserved protein kinase complex that integrates mitogenic cues such as growth factors, amino acids, and energy (ATP) levels to drive anabolic pathways such as protein translation (via phosphorylation of S6 kinase and 4E‐BP1), lipid and nucleotide biosynthesis and organelle biogenesis [32, 33, 34]. Activation of mTORC1 is controlled by nutrient availability (via the amino acid‐sensitive Ragulator‐Rag GTPase complex), and it is inactivated during starvation to allow a catabolic rather than anabolic metabolic state [32]. In senescent cells, however, mTORC1 signalling is observed to be constitutively activated, regardless of the fed or starved state, and fails to show nutrient‐dependent changes in either its activity or subcellular localisation (between the lysosomal membrane, where mTORC1 is active, and the cytoplasm) [27, 35]. mTORC1 activity is a key driver of the SASP and is implicated via several signalling pathways to contribute to the production of cytokines (prototypical examples include IL‐6, IL‐8) and matrix metalloproteinases (e.g., MMP‐1, ‐3) to support immune cell recruitment and matrix remodelling [19, 36, 37, 38, 39, 40]. mTORC1‐dependent inhibition of 4E‐BP1 enhances global protein synthesis but also specifically drives MAPKAPK2 translation. MAPKAPK2 binds the RNA‐binding protein ZFP36L1, which would otherwise degrade many SASP component mRNAs [17]. Additionally, mTORC1 drives SASP component transcription via NF‐κβ, a major stress‐responsive transcription factor responsible for the expression of many genes, including the inflammatory cytokines in the SASP (such as IL‐6, IL‐8, and CXCL1) [41, 42]. This occurs both directly, via the interaction of mTORC1 with the NF‐κβ regulator IKKα and indirectly, via mTORC1‐dependent translational upregulation of the cytokine IL‐1A. IL‐1A in turn enhances NF‐κβ activity via the regulator IKKβ [31, 43].

Mitochondria are intimately linked to senescence and indeed both an increase in mitochondrial content and increased mitochondrial dysfunction are observed in senescence. Increased Reactive Oxygen Species (ROS) production by dysfunctional mitochondria are reported to trigger a DDR and are implicated in both the acquisition and maintenance of the senescence program [44]. In a complex feed‐forward loop, the initial DDR has been shown to activate mTORC1, and drive mitochondrial biogenesis in a PGC‐1β‐dependent manner (Fig. 1A). The subsequent increase in mitochondrial content potentiates ROS production, inducing further DNA damage foci and driving a persistent DDR [15]. Several studies have implicated mitochondrial dysfunction in supporting the SASP through increased ROS [15], mitochondrial membrane damage, mtDNA leakage [45], and dysfunctional mitochondrial ROS production has also been linked to the presence of cytoplasmic chromatin fragments (CCF) [46]. Mitochondrial clearance in these cells has been shown to dampen several senescent phenotypes, including the inflammatory SASP [44, 46]. Indeed, forced degradation of mitochondria, via autophagy (a process called mitophagy) in senescent cells has been shown to reduce the senescent phenotype through reactivation of p53 [46].

The central role of mTORC1 signalling in the senescence programme and ageing is well established with dozens of studies demonstrating that mTORC1 inhibition by rapamycin slows acquisition of senescence *in vitro* and *in vivo* [29, 30, 31], including in the case of UVB‐induced senescence [47] and furthermore, rapamycin is one of the most potent interventions to improve lifespan in all model organisms tested. Even transient and late‐in‐life administration of rapamycin can extend the lifespan of some mouse models [48, 49]. Importantly, rapamycin treatment also extends the healthspan of mice, as measured by increased grip strength, age‐related weight gain, improved motor function, and reduced cancer incidence [49, 50]. Further to this, in humans, Chung *et al*. [51] showed that 10 μm topical rapamycin treatment given to an adult cohort aged > 40 years over 8 months reduced p16‐positive cells in the skin and the signs of ageing (as measured by clinical analysis of fine wrinkles, dyspigmentation, and the Merz Hand Grading Scale).

### The autophagy‐lysosome pathway in senescence

Autophagic flux, i.e. the formation of autophagosomes and their subsequent fusion with lysosomes, has been comprehensively shown to be intact in all senescence models tested, and in some cases increased [16, 27, 52, 53]. Chaperone‐mediated autophagy (CMA), whereby cargo are delivered directly to the lysosome via the resident protein Lamp2A, is also upregulated in several senescence models and cell types [53]. Furthermore, selective autophagy (p62‐ and NBR1‐dependent), along with CMA have been shown to be elevated, temporally later than increased bulk autophagy, in CDK4/6 inhibitor‐induced senescent melanoma cells [53]. Autophagy is necessary and sufficient for the induction of senescence by oncogenic Ras, specifically for survival during mitotic transition [52, 54]. Furthermore, spatial coupling of mTOR and auto‐lysosomes is observed in senescence, in a Rag‐GTPase‐dependent manner, and observed to correlate with SASP component translation (IL‐6/8) [27]. It is proposed that auto‐lysosomal degradation of autophagy cargo and liberation of amino acids supports mTORC1 activity [34] allowing mTORC1 to remain active on the lysosomal membrane. Autophagy is however a notoriously pleiotropic mechanism, and indeed the systemic loss of autophagy (which is widely noted in ageing) has also been shown to promote senescence and accelerate ageing in mouse models [55, 56].

Both autophagy and lysosome genes are upregulated in multiple forms of senescence at the transcriptional level, both dependent and independently of the transcription factors, TFEB/TFE3 [16, 27, 35, 52, 53], thus making lysosome biogenesis a robust hallmark of senescence (Fig. 1A). Our lab recently identified that the transcription factors TFEB/TFE3 show constitutive localisation to the nucleus, driving the biogenesis of lysosomes and expression of autophagy genes such as LC3 and p62 [16]. Interestingly, our work also indicates that individual lysosomes show less degradative capacity in senescent fibroblasts compared to proliferating controls, consistent with evidence that senescent lysosomes display increased pH [16, 57, 58] and signs of membrane damage (observed by galectin‐positivity) [16, 59]. We hypothesise that TFEB/TFE3 are activated as a compensatory mechanism to ensure maintenance of global cellular degradative capacity (which is remarkably similar to proliferating controls), and to support pro‐survival autophagy.

Dysfunction of the autophagy‐lysosome pathway is not only a hallmark of senescence but could crucially represent a targetable weakness in senescent cell metabolism. Inhibition of autophagy in senescent fibroblasts can cause cell death in the absence of exogenous nutrients, highlighting the importance of catabolism‐derived nutrients for senescent cell survival [35]. Damaged lysosomes cause metabolic alterations that can be targeted to promote senescent cell death. For example, Johmura *et al*. showed that lysosomal damage in senescent fibroblasts was associated with a reduction in cytosolic pH and a resultant expression of low pH‐induced kidney‐type glutaminase (KGA) which subsequently led to enhanced ammonia production to neutralise the pH and promote survival. Inhibition of KGA in aged mice was shown to promote the elimination of senescent cells. Moreover, in response to the lysosomotropic and membranolytic agent, LLOMe, senescent cells show increased cell death indicating that lysosomal membranes may be compromised and could be a senolytic target [59]. Finally, lysosomal expansion and dysfunction can lead to exocytosis, a process that is associated with increased lysosomal stress. Lysosomal exocytosis has been observed to be enhanced and contribute to the SASP *in vitro* in senescent melanoma and neuroblastoma cell lines [53, 60]. Indeed, the SASP contains many lysosomal proteins, and when lysosomal exocytosis is impaired via knockdown of RAB27a, there is a significant reduction in lysosomal components in the SASP from senescent melanoma cells [53]. Thus, there is increasing evidence that lysosomal expansion and dysfunction supports the senescence program and SASP via multiple pathways, contributing to cellular and tissue ageing, further reviewed in exceptional detail in Tan and Finkel [61].

## Senescence in the skin

**Fig. 2:**
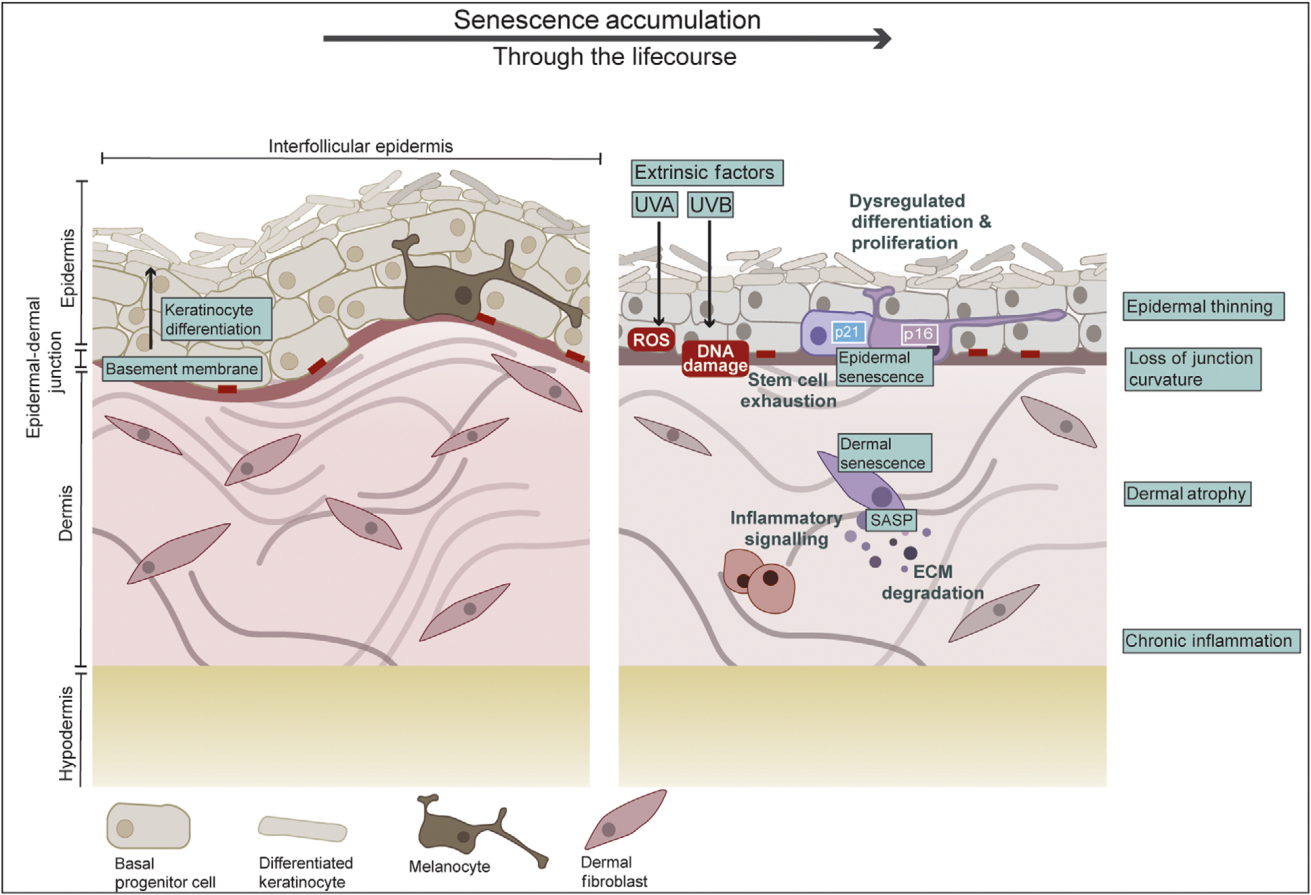
Impact of senescence on the ageing skin. The architecture of the skin consists of a stratified epidermis with terminally differentiating keratinocytes arising from a population of basal progenitor cells, interspersed with dendritic melanocytes. The epidermis is anchored by a basement membrane, below which is the mechanically strong dermis, dermal fibroblasts being the cell type responsible for extracellular matrix (ECM) deposition within this layer. With age, several phenotypic changes occur, including dysregulated epidermal differentiation, epidermal thinning, epidermal‐dermal junction flattening, dermal atrophy, and chronic inflammation. Extrinsic stressors, such as ultraviolet (UV) irradiation can drive senescence in the skin via DNA damage (induced by UVB irradiation) and oxidative damage (induced by reactive oxidative species (ROS) production, predominantly via UVA irradiation). Senescence has been identified in all major skin cell types: keratinocytes [62], melanocytes [18], and dermal fibroblasts [63], often marked by their positivity for expression of cyclin‐dependent kinase inhibitors (CDKIs), e.g., p16 and p21. Senescence has been shown to contribute to these age‐related changes, particularly via their deleterious secretory profile (SASP), which can dysregulate homeostasis within and between tissue layers. Specifically, the SASP can drive inflammation through cytokine expression and ECM degradation via matrix metalloproteinase expression [64, 65].

Ageing of tissues and organs is characterised by their loss of function and regenerative capacity. The skin is a large and complex organ providing a barrier to the external environment, essential in maintaining organismal health. The outermost layer is a stratified epidermis, below which is a thick, mechanically strong, and elastic dermis that sits atop an adipocyte‐rich hypodermis (Fig. 2). The major cell types within the skin possess distinct phenotypes and roles: the epidermis consists of a basal layer with long‐lived progenitor cells and melanocytes, and above the basal layer are keratinocytes undergoing terminal differentiation, regularly sloughing off. In the dermis, the tissue is largely acellular with dermal fibroblasts representing the major cell population. For all these cell types, age‐associated senescence accumulation has been identified, using the hallmarks of senescence such as CDKI expression, Sen‐β‐Gal staining, DDR markers, and the loss of LaminB1 [12]. While senescence is observed in the skin, and strongly correlates with ageing, the mechanistic detail underlying how senescence contributes to tissue ageing has not been extensively explored, particularly in regards to mTORC1 signalling and the autophagy‐lysosome pathway.

### Architecture of the skin

The epidermis is at the forefront of the skin's role as a barrier towards the external environment [66]. The epidermal basal layer contains proliferating progenitor cells, attached to the basement membrane via hemidesmosomes [67, 68, 69]. When cells detach from the basement membrane they differentiate, secreting waterproofing lipids and flattening into keratinocytes which are tightly connected via desmosome junctions [66, 68]. As keratinocytes reach terminal differentiation they fill with keratin and de‐nucleate [70, 71], dying in the *stratum corneum* (the outermost epidermal layer) and sloughing off [67]. Amongst the basal epidermal cells reside the melanocytes which are dendritic cells responsible for transferring melanin to keratinocytes to confer UV protection to the epidermis (Fig. 2) [72]. The interfollicular epidermis (IFE; see Fig. 2) is also interspersed with hair follicles (HFs), sweat, and sebaceous glands – which derive from the epidermis but descend into the dermis during development. HFs are rich in stem cells and highly proliferative, but these stem cells do not contribute to normal keratinocyte turnover in the IFE [73, 74, 75].

Below the epidermal‐dermal junction (EDJ) basement membrane (BM) resides the dermal tissue, the largest tissue layer in the skin, providing mechanical strength and nutrients to the epidermis. The dermis is primarily an acellular tissue, consisting of an extracellular matrix network of collagen and elastin to provide elastic absorption and load‐bearing properties to the skin [76]. Dermal fibroblasts are the dominant dermal cell type, their key role being ECM deposition (Fig. 2)—they differ in their secretory phenotype between the upper and lower layers of the dermis, with altered collagen and matrix metalloproteinase secretion to remodel/maintain the ECM architecture [77, 78]. Other cells and structures residing in the dermis include mast cells, blood vessels, and nerve endings. Below the dermis resides the fat‐rich hypodermis.

Exposure of skin to the Sun's damaging UV radiation is a major extrinsic factor driving ageing, although other important factors include pollutants and smoking [79]. The sun's UV radiation reaches Earth at different wavelengths that cause skin damage in different ways: UVA (315–400 nm) damages cells through an elevation in oxidative damage whereas UVB irradiation (280–315 nm) damages DNA directly [80]. Intrinsic ageing is associated with fine wrinkles, thinning of the epidermis, flattening of the EDJ, and dermal atrophy [81, 82, 83, 84, 85]. Extrinsic ageing, on the other hand, is restricted to areas exposed to the environment such as the face and forearms, and manifests as a thickening of the epidermis, dyspigmentation (including hyperpigmented macules), and deep wrinkles due to loss of elasticity and collagen in the dermis and EDJ [79, 86]. The stressors that drive extrinsic ageing also induce senescence, such as elevated ROS levels (as observed *in vitro* in naturally aged human dermal fibroblasts [87]) and UV‐induced DNA damage, making the role of senescence in skin ageing particularly important. Indeed, UVA/B irradiation have been shown to induce senescence *in vitro* in all major skin cell types: keratinocytes, melanocytes, and dermal fibroblasts [88, 89]. Aged skin has also been observed to show low‐grade chronic inflammation, with persistent immune cell recruitment, a hallmark of ageing often referred to as “inflammageing” (Fig. 2) [23].

### Senescence in the epidermis

#### Keratinocytes

Keratinocytes are a transient cell type arising from a population of progenitor cells residing in the basal layer of the epidermis. Senescence markers have been shown to accumulate with age *in vivo* in keratinocytes, including p21 [18], and the histone variant, H2AJ [62, 90]. However, p16, another CDKI commonly associated with senescence, has not been specifically observed in senescent keratinocytes *in vivo* [18], although it has been observed *in vitro* [91, 92]. Although p16 and p21 are largely considered interchangeable senescence markers, it is worth noting that a pre‐print transcriptomics paper from Saul *et al*. [93] reports that p21‐positive cells are the dominant age‐associated senescent population in both the dermis and epidermis and that the SASP of senescent skin cells are distinct between p16‐ and p21‐positive senescent cells. The exact mechanisms behind the distinct SASPs however remain to be elucidated. An age‐dependent increase in H2AJ‐positive cells has been identified throughout the epidermal strata but is most striking in the basal cell layer. H2AJ‐positivity correlates with TAFs and a loss of proliferative capacity [62]. As the basal layer contains proliferating keratinocyte progenitor cells, it could be hypothesised that an accumulation of senescence within this layer could impair keratinocyte cell turnover. This idea is supported by the observed age‐related epidermal thinning and loss of curvature (due to a loss in surface area) (Fig. 2) [94].

Lower circulating IGF‐1 levels have been found to correlate with extended lifespan in animal models, and it has also been linked, albeit controversially, to longevity in humans [95, 96, 97]. In the skin, IGF‐1 is thought to be secreted by dermal fibroblasts and signal via its cognate receptor, IGF‐1R on keratinocytes [98]. *In vitro*, IGF‐1 signalling has been reported to be key to determining keratinocyte fate in response to UVB irradiation. Specifically, exogenous IGF‐1 promotes UVB‐induced senescence and cell survival, whereas the absence of IGF‐1 leads to continued keratinocyte proliferation and apoptosis [94, 99]. Interestingly, IGF‐1 expression is lost when fibroblasts undergo senescence *in vitro* [100], and although it remains to be tested *in vivo*, these data highlight potential repercussions for keratinocyte fate and skin health as we age [99].

Keratinocyte differentiation is regulated via a complex set of factors including hemidesmosome degradation [69], reduced mTORC1 activity (and concomitant increased autophagy) [101], and calcium signalling [102]. Indeed, evidence from *in vitro* models suggests that the calcium gradient present in the epidermis [103, 104] may drive Ca^2+^‐dependent endoplasmic reticulum (ER) stress in differentiating keratinocytes. This ER stress was found to drive the unfolded protein response, ultimately triggering lysosome biogenesis and autophagy to facilitate differentiation [101, 105, 106]. To this point, pharmacological inhibition of ER stress or autophagy is reported to diminish calcium‐dependent differentiation [105, 106], while activation of autophagy (by mTORC1 inhibition) increase differentiation rates to 100% in culture [105]. Observations *in vivo* have shown that extrinsic ageing of the skin is associated with a loss in autophagic flux, particularly in the suprabasal strata of the epidermis, correlating with dysregulated epidermal proliferation, hyperpigmentation, and decreased epidermal barrier integrity [107]. Furthermore, expression of a mutant hemidesmosome component, COL17A1 that cannot be cleaved (a key step facilitating keratinocyte differentiation), is associated with increased Akt‐mTORC1 activity [108]. As mTORC1 and the autophagy‐lysosome pathways are altered in senescent cells, these aspects of epidermal homeostasis may be impacted by the accumulation of senescence, as discussed in the final section of this review.

#### Melanocytes

Melanocytes are specialised dendritic cells filled with melanosomes, lysosome‐related organelles that are the sites of melanin synthesis and storage. These melanosomes are transferred to keratinocytes to confer protection against UV irradiation across the epidermis [72]. Senescent melanocytes most notably contribute to melanocytic nevi, commonly known as moles, which are clusters of oncogene‐induced senescent melanocytes [103]. While nevi do not increase with age, melanocyte senescence does show an age‐dependent increase. Waaijer *et al*. and Victorelli *et al*. reported that the dominant population of p16‐positive cells in the epidermis are melanocytes [18, 104]. The p16‐positive melanocytes identified by Victorelli *et al*. in aged human skin also possessed length‐independent telomeric DNA damage foci (i.e. not replicative exhaustion). To identify key regulators of melanocyte senescence, the authors analysed *in vitro* melanocyte SASP and identified the cytokine, IP‐10. They showed that the IP‐10 receptor, CXCR3 is elevated in *in vivo* and *in vitro* senescent melanocytes and that the IP‐10‐CXCR3 axis plays an important autocrine role in melanocyte senescence. Crucially, IP‐10 also induces paracrine senescence in neighbouring keratinocytes and dermal fibroblasts. IP‐10‐CXCR3 signalling induces senescence via elevated mitochondrial ROS production, which in turn induces telomeric DNA damage foci (Fig. 1B). Epidermal thickness was rescued in three‐dimensional epidermal equivalents when senescent melanocytes were cleared via the senolytic drug ABT737 or mitochondrial ROS production was alleviated by the antioxidant, MitoQ. Interestingly, IP‐10 treatment was also reported to enhance Akt and ERK (1/2) phosphorylation in dermal fibroblasts, indicating the mTORC1 axis may also be impacted by this melanocyte SASP factor [18], although this has yet to be formally tested.

### Senescence in the dermis

Senescent dermal fibroblasts are observed to accumulate with age in human and primate skin, as identified by p16‐ and p21‐positivity *in vivo* [7, 63, 109] which correlates with age‐related morphological changes, such as disorganisation of elastin fibres and skin elasticity, and wrinkle formation [84]. The incorporation of senescent dermal fibroblasts into three‐dimensional organotypic skin equivalents leads to the development of several hallmarks of skin ageing such as epidermal thinning, a reduction in keratinocyte differentiation, and a reduction in barrier integrity [64]. Pharmacological induction of senescence in three‐dimensional skin equivalents also leads to a decrease in collagen and elastin fibres, linking senescence to age‐associated dermal atrophy [65]. Other work conducted in skin equivalents by An *et al*. corroborates these findings and further showed that there is an increase in MMP‐1 expression by senescent dermal fibroblasts, offering a mechanistic link between dermal senescence and atrophy [110]. Interestingly, this study found that pharmacological inhibition of 3‐phosphoinositide‐dependent protein kinase 1 (PDK1) reverses dermal fibroblast senescence and rescued the aged phenotype in skin equivalents [110]. PDK1 is an activator of Akt, which in turn can activate mTORC1. The PDK1‐Akt axis has previously been implicated in fibroblast senescence: *in vitro*, hyperactivation of Akt signalling via knockdown of PTEN (phosphatase and tensin homologue) in fibroblasts, for example, leads to premature senescence through stabilisation of p53 by mTORC1 and mTORC2, driving cell cycle arrest [111].

As aforementioned, activation of autophagy facilitates senescence in numerous cell types and senescence models. Indeed, UVB‐induced dermal fibroblast senescence leads to activation of autophagy and impairment of proteasomal degradation. This autophagic activation was partly in response to elevated ROS levels in these cells. Genetic inhibition of autophagy in these cells shifts cell fate from senescence to apoptosis upon UVB irradiation. Further to this, three‐dimensional skin equivalents treated with UVB results in elevated expression of autophagy and lysosomal markers in the dermis [54]. This study indicates that a prevalent exogenous stress, UVB irradiation, induces senescence via autophagic activation in the dermis. Interestingly, there is evidence that urban particulate matter impairs autophagy in UVB‐irradiated dermal fibroblasts and shifts cells towards apoptosis, indicating that naturally occurring insults interplay in determining cell fate [112].

In naturally aged dermal fibroblasts, a skin ageing‐associated secreted protein (SAASP) profile has been identified, independent of senescence, by Waldera Lupa *et al*. [113]. The SAASP and SASP share many similarities, including an upregulation in chemokines, cytokines and MMPs that may enhance the inflammatory and atrophic environment of the aged dermis. These analyses only included naturally aged cells that were still proliferative, however, it is important to consider the potential impact of pre‐senescent fibroblasts in the culture and the paracrine impact of any senescent fibroblasts present in the culture but excluded from the final analysis. Regardless, an inflammatory and ECM‐degrading secretory profile is present in the aged dermis which may be implicated in the age‐associated dermal atrophy observed.

#### Senescence and dermal atrophy

Age‐associated atrophy of the dermal ECM is associated with a reduction in Collagen types I and III, and a decrease in fibril thickness [114, 115]. Elevated MMP‐1 and ‐3 levels in the SASP (and SAASP) could alter the dermal architecture, as they degrade collagen I fibrils [39, 113]. The classical SASP is also associated with a decrease in tissue inhibitor of metalloproteinase 1 (TIMP‐1) expression, enhancing the impact of SASP‐associated MMP activity [39]. TIMP‐1 expression is also reduced in the aged and UV‐damaged dermis, and its expression is required to protect the dermis against ECM degradation and loss of elasticity [116]. Thus age‐associated dermal atrophy and reduction in elasticity could be reasonably linked to the ECM‐degrading SASP observed in dermal fibroblast senescence. Finally, the decellularization associated with age‐dependent dermal atrophy [85] may be impacted by the accumulation of senescent cells in the dermis due to the loss of their proliferative capacity. ECM degradation is also crucial in the regulation of epidermal proliferation, differentiation and cell turnover and is thus impacted by senescence in the dermis, as discussed later in this review.

A mouse model null for the cell surface collagenolytic enzyme MT1‐MMP (*Mmp14*) showed a more severe senescent load in several tissues, including the skin. *In vitro* analysis confirmed that this senescence induction was due to alterations in ECM remodelling due to the lack of MT1‐MMP activity. This study emphasises that not only can senescence impact the ECM, but that changes in ECM can also impact the induction and/or survival of surrounding senescent fibroblasts [117]. These senescent fibroblasts showed increased TGF‐β levels, a cytokine associated with paracrine senescence (and UVB‐induced senescence of human dermal fibroblasts [118]). Thus, understanding how the ECM can influence and amplify the senescent load in tissues is crucial. Interestingly, the loss of MT1‐MMP also impaired adipose tissue, including hypodermis thickness. Hypodermis thickness and function are essential to skin health and appearance. Reducing the senescent load in these mice via treatment with retinoic acid rescued the loss in hypodermis thickness and extended the lifespan of the mice [117], presenting retinoic acid as a potential senotherapeutic.

#### Dermal senescence and inflammation

The inflammatory components of the SASP have been proposed to drive the chronic, low‐grade inflammation associated with age (termed “inflammageing”). Inflammageing is associated with the activation of the innate immune system by inflammatory cytokines and an increase in the ratio of CD4+: CD8+ T‐cells in aged tissue, including skin [21, 23, 119]. The cytokines present in the SASP are capable of recruiting and activating both innate (e.g., natural killer cells, macrophages [8, 9]) and adaptive (e.g., CD4+ T‐lymphocytes [9]) immune cells, to drive senescence clearance. However, age‐related loss in immune system competency may compromise senescence clearance, which is exemplified by the increase in senescent cells observed within the dermis of mouse models lacking immune cell cytotoxicity [10]. This failure to remove senescent cells from the dermis may subsequently contribute to increased inflammatory SASP. Furthermore, senescent dermal fibroblasts *in vitro* have been observed to secrete extracellular vesicles with increased IL‐6 levels, another inflammatory cytokine that may impact surrounding tissue in the aged dermis [120]. Finally, ECM‐degrading components in the SASP could facilitate inflammation by increasing accessibility for immune cell infiltration.

In dermal fibroblasts specifically, there is evidence that senescent cells avoid clearance via several immune‐dampening strategies. This may further drive the age‐related accumulation of senescent cells in the dermis. Firstly, senescent dermal fibroblasts in culture are observed to increase expression of the major histocompatibility complex HLA‐E, which reduces attack by natural killer and CD8+ T‐cells (Fig. 1B). This is corroborated by *in vivo* analysis of aged skin [22]. *In vitro* work by Ogata *et al*. [121] also shows that pre‐treatment with SASP components reduces the ability of macrophages to kill dermal fibroblasts, with factors such as IL‐1α and GM‐CSF impairing their phagocytic capabilities. Finally, work by Narzt *et al*. [122] showed that lysophosphatidylcholines are upregulated in dermal fibroblast SASP and also impair macrophage phagocytic activity.

The inflammatory factors of the fibroblast SASP have been shown *in vitro* to have an autocrine role in maintaining cell cycle arrest [42], as well as paracrine roles in inducing neighbouring cell senescence, which is corroborated by *in vivo* studies [123]. In dermal fibroblasts, it remains an interesting open question whether paracrine signalling is responsible for the overlap in inflammatory components between fibroblast SASP and the SAASP (e.g., IL‐8, IL‐1β, and IFN‐γ [7, 8]).

Lysosomal membrane integrity and composition are linked to inflammatory signalling, for example, lysosomal damage and Cathepsin B leakage can activate the inflammasome by acting through NLRP3 signalling [124]. Additionally, recent analysis of senescent cell metabolism by Roh *et al*. revealed that cholesterol homeostasis‐ and fatty acid metabolism‐related genes are amongst the most altered, this led to the identification that the SASP is enhanced by cholesterol accumulation at the lysosomes of senescent dermal fibroblasts. Specifically, lysosomal cholesterol accumulation, due to repurposing of the cholesterol exporter ABCA1 as a lysosomal importer, supported the constitutive activation of mTORC1 [125]. mTORC1 activity then subsequently drives the expression of SASP factors, such as IL‐1A, IL‐6/‐8, and MMPs [31, 125]. Further to this, the most increased gene expressions in intrinsically aged dermal fibroblasts are those involved in lipid metabolism, including cholesterol metabolism, and the innate immune system [126]. Understanding how lipid metabolism is dysregulated in senescence, how it impacts (and is affected by) the immune system, and what the metabolic consequences are for senescent cells will help understand the mechanisms driving dysfunction of aged tissue.

### Cell crosstalk and future perspectives

#### Melanocyte‐keratinocyte crosstalk

Melanin‐synthesising melanocytes decrease with age, and increase in heterogeneity, leading to mottled skin pigmentation and impaired skin defence against UV radiation [127, 128]. Melanocyte‐keratinocyte crosstalk has already been touched on, and there is evidence that mTORC1 signalling could play a role in this crosstalk. For example, hyperactive mTORC1 signalling in patients with tuberous sclerosis correlates with loss of pigmentation in hair follicles (and early hair greying), as well as hypopigmented skin macules [129, 130]. Another study identified that inhibition of mTORC1 activity in keratinocytes rescues melanocyte function in grey hair follicles through rescued expression of the peptide hormone, α‐melanocyte stimulating hormone (α‐MSH), which drives melanocyte dendricity, melanogenesis, and melanosomal transfer. Crucially, mTORC1 inhibition in *ex vivo* human tissue rescues melanocyte function [131]. While speculative, this raises the possibility that an accumulation of senescent cells, with constitutively active mTORC1 activity, in the epidermis, may negatively influence melanocyte function, potentially altering skin pigmentation, and contributing to age‐related skin changes such as hypopigmentation. Senescent dermal fibroblasts however have also been implicated, both *in vitro* and *in vivo*, with localised ageing‐associated hyperpigmentation (lentigo). More specifically, a decrease in stromal cell‐derived factor 1 (SDF‐1) in the dermal fibroblast SASP has been implicated in driving excessive melanogenesis [132], leading to increased melanin production. Clearly, more research is required to fully establish how induction of senescence in different cell types influences skin pigmentation heterogeneity.

#### Keratinocyte differentiation and epidermal integrity

Chronological, or intrinsic ageing is associated with epidermal thinning, with changes in the rate and efficiency of keratinocyte differentiation being widely implicated. Whether and to what extent senescence contributes to this phenotype is not well understood. Several studies indicate direct and/or indirect effects of senescence on the integrity of the epidermis. Although not formally tested, the characteristic simultaneous activation of the autophagy‐lysosome pathway and mTORC1 signalling observed in senescence [33] could directly impact basal epidermal cell fate. Keratinocyte differentiation is associated with a loss of mTORC1 and the concomitant increase in autophagy; it could therefore be hypothesised that an increase in senescence in the basal epidermal cells could abrogate differentiation due to persistent mTORC1 signalling [105], contributing to epidermal thinning. Furthermore, this may extend the residency of senescent cells in the epidermis, prolonging the impact of their inflammatory and deleterious SASP, particularly as immune clearance of senescent cells declines with age [10].

There is evidence that increased accumulation of senescent cells in the dermis may disrupt Collagen type XVII (COL17A1)‐regulated keratinocyte differentiation pathway. COL17A1 is a transmembrane protein localised to hemidesmosomes that is important for anchoring the actin cytoskeleton of basal keratinocytes with the ECM, thus anchoring the epidermis to the dermis. MMP‐dependent cleavage of the COL17A1 ectodomain is an important initial step in the terminal differentiation of keratinocytes (Fig. 1B) [81, 82]. In both mouse models and humans, COL17A1 levels have been widely observed to decrease with age which has important and complex implications for epidermal health. The age‐related loss of COL17A1 in basal epidermal cells is not uniform and while those cells with low COL17A1 levels will be lost via terminal differentiation, those with persistent COL17A1 expression are reported to subsequently undergo clonal expansion through symmetrical cell divisions at the basement membrane, leading to a loss in heterogeneity and eventual exhaustion of the remaining stem cell population. These changes are observed to ultimately lead to a thinning of the epidermis [20]. Importantly, exposure of skin to genotoxic stress and UV irradiation in mouse models leads to enhanced MMP‐dependent COL17A1 degradation [20]. Dermal fibroblast senescence is associated with the secretion of several MMPs as part of the SASP and indeed, three‐dimensional skin equivalents prepared with senescent dermal fibroblasts show decreased COL17A1 levels and reduced epidermal thickness [110]. It remains to be formally tested whether these effects could be prevented or reversed by MMP inhibitors or modulation of expression of fibroblast SASP factors, but it's likely that SASP‐associated proteases impact keratinocyte differentiation and contribute to accelerated skin ageing.

In addition to the paracrine impact of the fibroblast SASP on keratinocytes, senescent dermal fibroblasts have also been reported to upregulate EV biogenesis and secretion. *In vitro* treatment of keratinocytes with these purified EVs leads to a significant reduction in expression of differentiation markers such as loricrin, as well as reduced expression of bleomycin hydrolase (involved in barrier function) and increased expression of the SASP factor IL‐6 (but not IL‐8) [120]. The authors propose that senescence‐associated dysfunction of lysosomes is an important regulator of EV secretion, and indeed dysregulating the lysosomes of control fibroblasts (by treatment with the V‐ATPase inhibitor, Bafilomycin A), increased EV secretion [120]. Further to this, lysosomal exocytosis is also enhanced in response to senescence‐inducing stressors such as UV irradiation [53, 60]. These observations are particularly interesting in the context of the epidermis where, as discussed above, lysosome‐dependent degradation is a key regulator of keratinocyte transition to terminal differentiation [133]. Taken together, it will be interesting to establish the mechanisms, the overlap, and the extent to which dysfunctional lysosomes, EV secretion and soluble SASP factors from dermal fibroblasts contribute to age‐related changes in skin and this could have important implications for the development of senotherapeutics. For example, our work, and others, identified that senescence is associated with lysosome biogenesis but that lysosomal membranes show increased signs of damage, thus, challenging senescent cells with lysosomotropic drugs promotes cell death. There is increasing molecular understanding of the numerous pathways in which lysosomal membrane damage can be repaired, including ESCRT complexes and transfer of lipids from the ER, amongst others. More work is needed to explore these mechanisms in senescence and specifically in senescent skin cells but they could represent novel targets for the development of senotherapuetic interventions.

## Conclusion

There is a clear correlation between the age‐related phenotypic and morphological changes in skin and the accumulation of senescent cells. The underlying mechanisms, however, are still to be identified, and we believe there is an important and underappreciated role for dysregulated mTORC1 signalling and the autophagy‐lysosome axis. The research reviewed here highlights the evidence that these pathways are both important in senescence acquisition and maintenance, and as sensitivities in the senescence program. Thus, they offer potential as targets to extend the healthy lifespans of an ageing population – as rapamycin has already been demonstrated in animal models. To drive the progress of this research area it is not only key to understand how mTORC1 activity and the autophagy‐lysosome pathway support the deleterious senescent phenotypes, such as the inflammatory and atrophic SASP, but also how they impact cell function in the context of cell and tissue crosstalk.

## Conflict of interest

The authors declare no conflict of interest.

## Author contributions

PS was involved in writing of manuscript and creation of figures. BC was involved in editing of manuscript.
